# Better after Sleep*Dr. Dunham said Hahnemann used “with great success the sixtieth, the one hundred and fiftieth, and the three hundredth dilutions.” Dr. Dunham also adds: “It is not unworthy of remark that as Hahnemann’s practical experience in the treatment of disease increased, so did his estimate of the advantage and necessity of using the higher dilutions, in at least many cases likewise increase.”The experience of those who so loudly decry high potencies would be similar to that of Hahnemann and Bœnninghausen did they prescribe with equal diligence and accuracy.

**Published:** 1882-12

**Authors:** C. Hering


### BETTER AFTER SLEEP.

Sleep, soon after the first dose of a well-chosen medicine often warrants the improvement of the patient. Feeling refreshed by sleep, particularly the mind, is a sure sign of improvement. Hence, we ought never to forget to ask the patient: "How do you feel when first wakening from sleep?" or the mother: "How is the child when waking of its own accord?"

Better after sleep is mostly an indication to leave the patient without further medicine. In rare cases only does it indicate a medicine.—C. HERING.

